# Extinction in Phylogenetics and Biogeography: From Timetrees to Patterns of Biotic Assemblage

**DOI:** 10.3389/fgene.2016.00035

**Published:** 2016-03-22

**Authors:** Isabel Sanmartín, Andrea S. Meseguer

**Affiliations:** ^1^Real Jardín Botánico, RJB–CSICMadrid, Spain; ^2^INRA, UMR 1062, Centre de Biologie pour la Gestion des Populations – INRA– IRD–CIRAD–Montpellier SupAgroMontferrier-sur-Lez, France

**Keywords:** diversification, birth–death models, global diversity patterns, speciation, mass extinction, asymmetric spatial extinction, likelihood-based methods, Bayesian inference

## Abstract

Global climate change and its impact on biodiversity levels have made extinction a relevant topic in biological research. Yet, until recently, extinction has received less attention in macroevolutionary studies than speciation; the reason is the difficulty to infer an event that actually eliminates rather than creates new taxa. For example, in biogeography, extinction has often been seen as noise, introducing homoplasy in biogeographic relationships, rather than a pattern-generating process. The molecular revolution and the possibility to integrate time into phylogenetic reconstructions have allowed studying extinction under different perspectives. Here, we review phylogenetic (temporal) and biogeographic (spatial) approaches to the inference of extinction and the challenges this process poses for reconstructing evolutionary history. Specifically, we focus on the problem of discriminating between alternative high extinction scenarios using time trees with only extant taxa, and on the confounding effect introduced by asymmetric spatial extinction – different rates of extinction across areas – in biogeographic inference. Finally, we identify the most promising avenues of research in both fields, which include the integration of additional sources of evidence such as the fossil record or environmental information in birth–death models and biogeographic reconstructions, the development of new models that tie extinction rates to phenotypic or environmental variation, or the implementation within a Bayesian framework of parametric non-stationary biogeographic models.

## Introduction

“Species and groups of species gradually disappear, one after another, first from one spot, then from another, and finally from the world.”

(Darwin, 1859)

At a time when nearly one tenth of species on Earth are projected to disappear in the next 100 years (Maclean and Wilson, 2011), extinction has become an important topic of research in biology (Purvis, 2008; Rabosky, 2010; Morlon et al., 2011; Stadler, 2011a,b; Beaulieu and O’Meara, 2015). Paleontologists have long been concerned with extinction and its effects on patterns of biotic assembly (Benton, 2009). The fossil record shows the footprint of several events of large-scale (mass) historical extinctions that have changed the composition of communities and biomes at global scale – the “Big-Five” – (Benton, 2009). The current biodiversity crisis is considered by many scientists as the next large-scale extinction event, in which human actions have caused accelerated rates of species extinction across many clades within a time span of 100s rather than millions of years (Barnosky et al., 2011). Inferring the effect of past extinction events on macroevolutionary dynamics and patterns of biotic assembly might be useful to understand current threats on present diversity, and thus mitigate their effects (Purvis, 2008). Yet, until recently, extinction has received little attention in macroevolutionary studies compared to speciation (Moore and Donoghue, 2007; Rabosky et al., 2007; Rabosky and Lovette, 2008; Rabosky, 2009). One reason for this is that extinction is by its very nature difficult to measure. Except for a few cases (e.g., viruses, recent catastrophic volcanic events), we cannot observe speciation or extinction at work because it takes 100s of years for a species to speciate or go extinct, so we need to infer these processes from present and historical data. However, while we can observe the outcome of speciation in extant taxa, an entire clade can go extinct without leaving a trace. Furthermore, if extinction rates have been asymmetric among clades or areas, the diversity patterns we observe today might be a poor representation of the historical diversification process (Morlon et al., 2011; Meseguer et al., 2015). Another reason for the prominence of speciation over extinction can be traced back to Darwin, who viewed extinction mainly as a process filtering the existing variation, the overproduction of offspring over which natural selection may act. Under the “Red Queen” model of Van Valen (1973), which stems from Darwin (Benton, 2009), evolution is driven by biotic factors such as interactions among species (e.g., competition, predation) or species-intrinsic biological traits (e.g., body size, ecological tolerances) increasing reproductive fitness. Extinction in this model acts gradually on individual clades. This view stands in contrast with the “Court Jester” model, prevalent among paleontologists (Barnosky, 2001; Benton, 2009), in which macroevolutionary dynamics (speciation and extinction) are the result of abiotic, extrinsic factors such as geological tectonic events or abrupt changes in climate, usually acting at longer time scales. In this model, extinction can simultaneously affect multiple clades (Benton, 2009). Recently, Ezard et al. (2011) proposed an intermediate model, in which speciation is modeled by species-intrinsic factors, while extinction is more dependent on abiotic factors acting clade-wide, across different groups of organisms.

Although the Red Queen model accepts also the influence of environmental change on evolution – species must evolve to keep pace with the changing environment or go extinct (Van Valen, 1973) – and Darwin regarded extinction as a key process in generating biotic patterns in his famous sketch in Notebook B (1837) of the Tree of Life, according to the Red Queen model, causes of extinction are primarily biological, rather than physical, with extinction acting continuously (the model assumes constant extinction rates) at the microevolutionary level of individuals, populations, and species. The Court Jester model (Benton, 2009), in contrast, introduced a more macroevolutionary and macroecological view to extinction, in which abrupt changes in the physical template like climatic or tectonic events could drive extinction rates at regional or global scale, e.g., mass extinctions (ME; Benton, 2009). For example, the fact that tropical regions in Africa are species-poor compared to those found in other continents in the same latitudes, such as South East Asia or the Neotropics (Plana, 2004; Kissling et al., 2012), has sometimes been attributed to historically higher extinction rates in this continent, due to an aridification trend that started in the Miocene (Senut et al., 2009; Couvreur, 2015).

The different roles conferred to extinction in the Court Jester and the Red Queen models are also relevant for the field of biogeography, the study of patterns of biodiversity distribution and their underlying ecological and evolutionary causes (Sanmartín, 2012). If extinction rates are driven by abiotic factors, as in the Court Jester model, they might be area-dependent, i.e., they could be associated to the spatial distribution of the clade. For example, Jablonski (2008) demonstrated that survivorship to ME events was positively correlated with the geographic range of taxa. A recent study (Meseguer et al., 2015) showed a correlation between a clade’s rate of extinction and its present and past biogeographic distribution. In addition, catastrophic (mass) extinction events that act across unrelated clades sharing the same area of distribution are expected to shape biogeographic patterns at the regional rather than at the local scale. If the change in the physical template is too rapid or large for species to adapt or migrate to more favorable areas, extinction and fragmentation of the original distributional range (vicariance) ensue (Wiens, 2004). For example, the existence of continental-scale biogeographic disjunctions in several lineages of non-tropical African plants, the “Rand Flora” pattern, has been linked to successive aridification waves driving high extinction rates that fragmented a former widespread distribution across northern and central Africa (Sanmartín et al., 2010; Pokorny et al., 2015). The Australian flora offers a similar case, in which the formation of the arid Nullavar Plain produced a congruent molecular signature of vicariance across multiple plant clades (Crisp and Cook, 2007).

From all this, it follows that extinction can be both a process acting on individual clades over time and an agent shaping biogeographic patterns across unrelated clades that share the same distributional range. Therefore, studies of extinction should benefit from the consideration of the two different perspectives, the temporal and the spatial (Jablonski, 2008), and the use of both macroevolutionary and biogeographic approaches. Here, we review different methods to infer extinction rates from temporal (the structuring of branches in a clade’s phylogeny) and spatial (the geographic range of clades in a biogeographic reconstruction) data. We analyze the effect of extinction on our ability to retrieve evolutionary history under different high extinction scenarios or when there is heterogeneity in extinction rates over time and/or across areas and clades. We also identify the most promising avenues of research in macroevolution and biogeography, which include: (i) the integration of additional sources of evidence, such as the fossil record or environmental information, in birth–death models and biogeographic reconstructions; (ii) the development of new models that tie extinction rates to phenotypic or environmental variation; or (iii) the implementation within a Bayesian framework of time-heterogeneous biogeographic models.

## Phylogenetic Approaches to the Inference of Extinction

### Inferring Lineage Extinction from Time Trees

**FIGURE 1:**
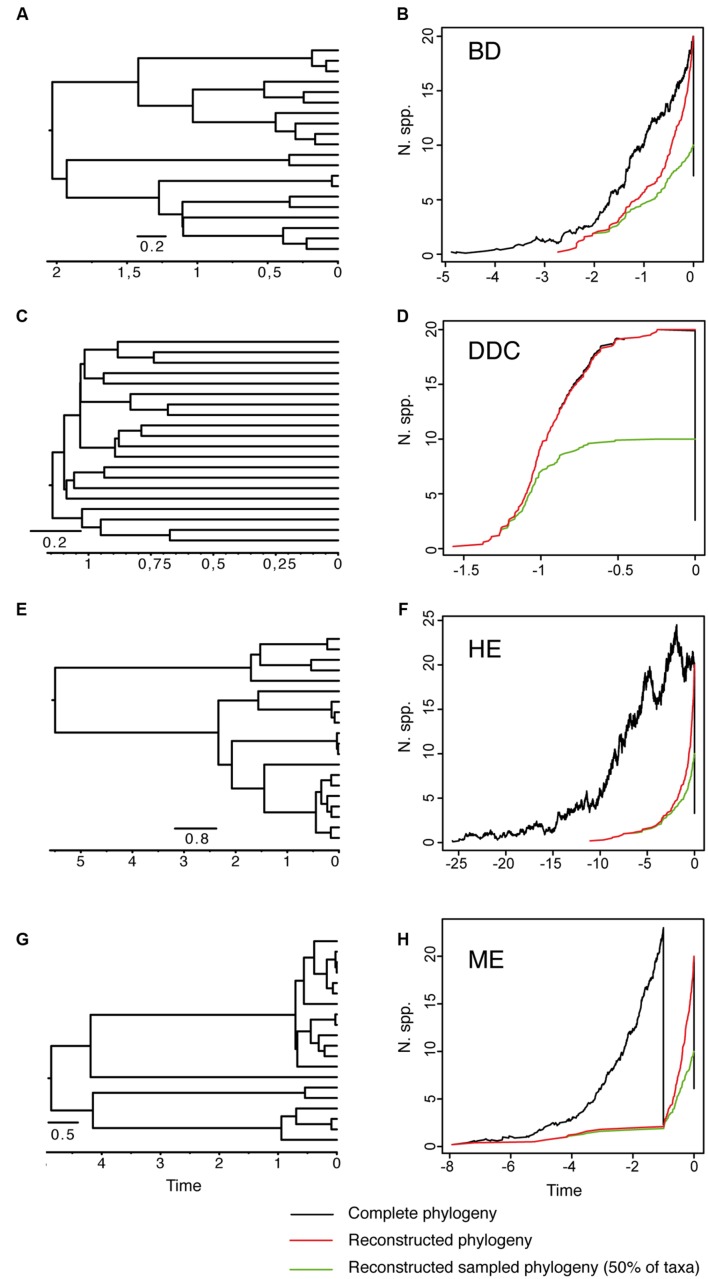
**Reconstructed phylogeny and lineage-through-time (LTT) plot of a 20-taxa phylogeny simulated under alternative birth–death models. (A,B)** Constant-rate Birth–Death model (BD) simulated under birth rate λ = 2, death rate μ = 1 and with extinction fraction *a* = 0.50. **(C,D)** Diversity-Dependent Cladogenesis model (DDC) simulated under a discrete approximation in which μ = 0.01 and speciation rate decreases exponentially through time λ(*t*) = *e* ^-^*^(K^*^t)^, with *K* = 5, λ_0_ = 6, using a time grid of 0.2 time units (e.g., λ = 0.04, 0.11, 0.29, 0.81, 2.21, 6). **(E,F)** High-background extinction model (HE) simulated under λ = 2, μ = 1.9 and *a* = 0.95. **(G,H)** Mass extinction model (ME) simulated as a constant-rate birth–death model with an initial period of rapid tree growth (λ = 2, μ = 0.3, *a* = 0.15) interrupted by a ME event at time *t* = 1 that removes 90% of lineages, and followed by a recovery period with slower growth (λ = 2, μ = 1, *a* = 0.50). Ten phylogenies were simulated for each model using the function *sim.bd.taxa* in the R package *TreeSim* (Stadler, 2011c) conditional on a number of 20 extant taxa (*N* = 20). For the ME model, we used the command *sim.rateshift.taxa* conditioning on the number of extant taxa and the time of the ME event using as sampling fraction ρ = 0.1. The phylogeny to the left **(A,C,E,G)** represents one of the 10 reconstructed phylogenies simulated under each model. The black line in the LTT plot represents the average LTT plot over the 10 simulated phylogenies for the “complete” birth–death process, i.e., including extant and extinct taxa (thus, the varying number of initial taxa), and using the option COMPLETE = TRUE; the red line shows the average LTT plot for the corresponding “reconstructed” phylogeny after removing the extinct lineages using the function *drop.extinct* in the R package *geiger* (Harmon et al., 2008). The green line in the LTT plot represents the reconstructed “sampled” phylogeny in which only 50% of the original extant taxa have been sampled (*N* = 10); the latter was done with a sample algorithm script that randomly removed tips from the reconstructed tree using the *drop*.*tip* function from the R package *ape* (Paradis et al., 2004).

As reviewed by Purvis (2008), early attempts at inferring lineage extinction rates were based on the fossil record – the turnover in fossil forms in consecutive, sequential stratigraphic data (Foote, 1988; Jablonski and Chaloner, 1994; Alroy, 2009). However, for most groups the fossil record is incomplete and often biased toward certain forms. The molecular revolution and the possibility to integrate time into phylogenetic reconstructions has brought about the possibility of inferring speciation and extinction rates from a “reconstructed” phylogeny containing only extant taxa (Nee et al., 1992, 1994; Harvey et al., 1994; Nee, 2006; Purvis, 2008). The basic idea is that speciation and extinction leave distinct signatures in the branching structure of these phylogenies (Nee et al., 1992). To illustrate this, we simulated 10 phylogenies under alternative speciation (birth) and extinction (death) models conditional on a final number of *N* = 20 extant taxa (**Figure 1**). **Figure 1A** shows a phylogeny generated under a model of diversification in which the rate of origination (speciation, λ) and extinction of lineages (μ) is kept constant over time. **Figure 1B** shows the corresponding lineage-through-time (LTT) plot, a curve depicting the accumulation of lineages over time. The black line represents the average LTT plot for the 10 “complete” phylogenies, i.e., if extant and extinct taxa are included in the phylogeny; the red line represents the average LTT plot of the corresponding “reconstructed” phylogenies, if only extant lineages that survived to the present are represented. In birth–death models, speciation and extinction are modeled as stochastic events that occur between waiting times of cladogenesis distributed according to an exponential distribution (Nee et al., 1992). Under the constant-rate birth–death (BD) model (**Figures 1A,B**), lineages in a reconstructed phylogeny accumulate through time with rate *r* = λ – μ, and accumulate in the very recent past with rate λ, i.e., because younger lineages had no time to go extinct. The change in rate of lineage accumulation or slope from λ–μ to λ is called the “pull-of-the-present” and allows estimating (separately) both λ and μ given only data from extant species (Harvey et al., 1994). Usually, one does not estimate birth and death rates separately, but instead estimates two indirect terms: the “net rate of diversification” (*r* = λ – μ), and the extinction fraction *a* = (μ/λ), also called “background extinction” or “species turnover.”

The extinction fraction, or the ratio of extinction to speciation, is responsible for the pull of the present and the difference between the reconstructed and the complete phylogeny. This can be seen in the density-dependent cladogenesis (DDC) model (**Figures 1C,D**), where the rate of diversification decreases exponentially as a function of the number of lineages until it reaches a plateau or equilibrium, at which point speciation equals extinction. In **Figure 1C**, speciation rates decrease exponentially and extinction was kept constant and close to zero (μ ∼ 0). There is no pull of the present so the LTT plot of the complete and reconstructed phylogenies offer a nearly perfect match (**Figure 1D**). The opposite effect can be seen in the high background extinction (HE) model represented in **Figure 1E**. Here, extinction rates are kept high relative to speciation, i.e., the extinction fraction is close to 1 (*a* = 0.95), so the pull of the present is very marked, giving the false impression of accelerating diversification rates toward the present in the LTT plot (**Figure 1F**). Branching times (diversification events) in the reconstructed phylogeny (**Figure 1E**) cluster toward the tips, leaving a so-called “handle-and-broom” shaped tree with long basal branches and bushy distal clades (Crisp and Cook, 2009). In comparison, the reconstructed phylogeny of the BD model, in which the extinction fraction is *a* = 0.5 (half of lineages that originate go extinct), shows a tree with more regularly spaced branching events (**Figure 1A**). This difference in tree shape caused by the extinction fraction or the pull of the present is best captured by the gamma statistics (γ, Pybus and Harvey, 2000), a measurement that compares the relative position of node ages in a phylogenetic tree with that expected under a pure birth model (Yule, 1924). Values lower than 0 (γ < 0) or higher than 0 (γ > 0) indicate, respectively, that internode distances are longer or shorter toward the recent than expected under the Yule model. Estimates of the gamma statistics for our simulated phylogenies (**Table 1**) show that values are highly negative in the DDC model (nodes tend to accumulate toward the root), but positive in the HE model (nodes tend to accumulate toward the tips), while in the BD model, some gamma values are close to zero. There is, however, stochastic variation and overlap in the range of gamma values across models (**Table 1**; see also the histograms in Supplementary Figure 1).

**Table 1:** Some statistics associated to the phylogenies simulated in **Figures 1** and **3** under alternative birth–death models.

Model	Sampling	Gamma Stat: Median	Gamma Stat 95% Confidence interval	N° extinct taxa: Mean (Standard deviation)
BD	Rec	1.40	(+0.48 to +2.32)	26.2 (7.38)
	Samp	-0.22	(–0.73 to +0.27)	
DDC	Rec	-4.99	(–5.31 to –4.67)	0.2 (0.63)
	Samp	-3.64	(–4.02 to –3.25)	
HE	Rec	1.83	(+1.16 to +2.49)	351.2 (260.5)
	Samp	0.40	(–0.60 to +1.41)	
ME	Rec	2.31	(+0.72 to +3.91)	31.1 (17.43)
	Samp	1.22	(+0.43 to +2.0)	
SRD	Rec	0.75	(+0.15 to +1.35)	0.9 (1.85)
	Samp	0.20	(–0.39 to +0.79)	

*BD: (birth–death model) a constant-rate birth–death model with a moderate extinction fraction (a = 0.50); DDC: (density-dependent cladogenesis model), a exponentially decreasing speciation model with low extinction; HE: (high extinction model), a constant-rate birth–death model with a high extinction fraction (a = 0.95); ME: (mass extinction model), a birth–death model interrupted by a ME (sampling event) that removes 90% of lineages at time t = 1 Ma; SRD: (stasis and rate-shift diversification model) described in **Figure 3** (see **Figure 1** for more details on simulations). Rec: statistics estimated for the “reconstructed” phylogenies with all extant species included; Samp: statistics estimated for the “sampled” phylogenies, in which only 50% of extant species were sampled. Gamma Stat: median and 95% confidence interval around median (Chambers et al., 1983) of the gamma statistic estimated over 10 trees simulated under each birth–death model (see Supplementary Figure 1 for histograms representing the variation in these values). Number of extinct taxa: mean and standard deviation for the number of extinct taxa in the “complete” phylogenies simulated under each model (see text).*

#### Effects of Incomplete Taxon Sampling and Episodic Mass Extinction on Birth–Death Models

There are several factors that make it difficult to estimate the extinction fraction from the shape of a reconstructed phylogeny or the change of slope in the LTT curve. One of the best studied is the effect of incomplete taxon sampling (ITS). It is not uncommon for reconstructed phylogenies to include only a small sample of the total number of extant taxa in a given clade, due, for example, to difficulties in obtaining samples for all taxa, poor quality of the extracted DNA, failure of the sequencing protocol for some markers, etc. ITS – analogous to the effect of extinction removing lineages at present time (*t* = 0 million years, Ma) – has the opposite effect to the extinction fraction on the LTT plot. It removes the pull of the present (see **Figures 1A–F**), resulting in reconstructed rates that are underestimated for the extinction fraction, i.e., the reconstructed phylogeny often fits a pure birth model with μ ∼ 0 (Pybus and Harvey, 2000). The green line in **Figure 1** represents the average LTT plot of the 10 reconstructed phylogenies under each model if only half of the extant species has been included (sampling fraction ρ = 0.5). In all models, the sampled phylogeny shows a more flattened LTT curve than the corresponding reconstructed phylogeny (**Figure 1**). In the DDC model (**Figures 1C,D**), the “sampled” LTT plot (green line) reaches the plateau or equilibrium carrying capacity earlier than in the reconstructed LTT (red line). Sampling was simulated as a random pruning of tips in **Figure 1**. However, in real phylogenies, sampling is often phylogenetically overdispersed, i.e., maximizing the representation of major clades in the tree. This has the effect that cladogenetic events closer to the tips (young clades) tend to be undersampled relative to basal clades, which explains why many empirical phylogenies – where taxon sampling often ranges between 20 and 80% – show a good fit to the DDC model (Cusimano and Renner, 2010). The effect of ITS on the pull of the present is shown also in the gamma statistic, where values tend to be smaller for the sampled phylogenies in comparison with the corresponding reconstructed (100% sampling) phylogeny (**Table 1**; Supplementary Figure 1).

Another source of bias in estimating the extinction fraction comes from the fact that different birth–death models can give similarly shaped reconstructed phylogenies and LTT plots (Quental and Marshall, 2010). Therefore, methods that evaluate diversity trajectories such as the gamma statistic can be sometimes misleading. For example, a phylogeny with branching times clustering toward the root can be generated by the DDC model in **Figures 1C,D**, which is often interpreted as the phylogenetic signal of an evolutionary radiation: species first accumulate rapidly and then increasingly slower as ecological niches are being filled by new species (Rabosky and Lovette, 2008). However, a similarly shaped phylogeny could be generated by a model with constant speciation rates and exponentially decreasing extinction rates, or by a model of exponentially decreasing speciation and exponentially increasing extinction rates (Rabosky and Lovette, 2008). Although these models may be distinguished using likelihood-based methods (Rabosky and Lovette, 2008), Quental and Marshall (2010) demonstrated that if the initial speciation rate is low compared with the extinction rate (the LiME ratio), a true diversification decline could not be inferred irrespective of the magnitude of the extinction rate.

**FIGURE 2:**
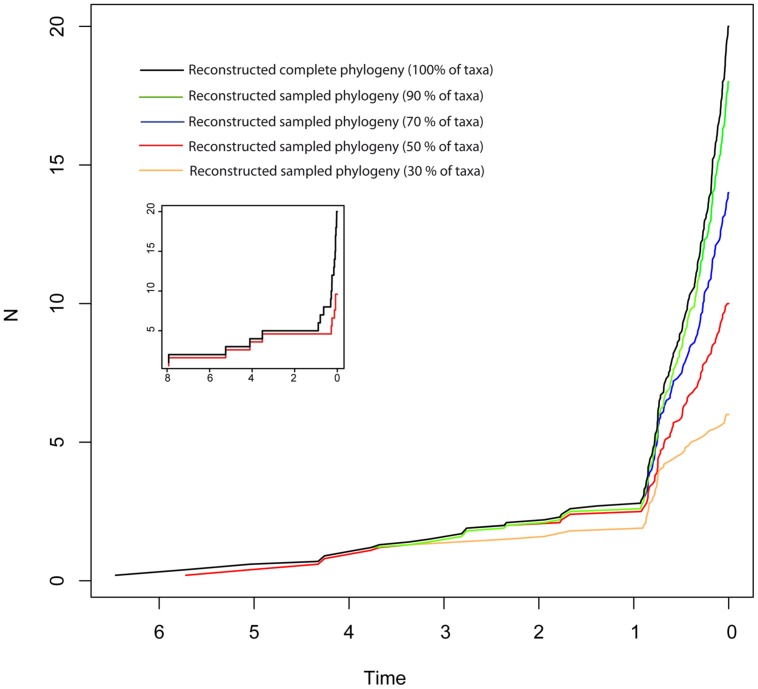
**Effect of incomplete taxon sampling (ITS) on the reconstructed LTT plot of the ME model (**Figures 1G,H**).** The black line shows the average LTT plot of the 10 reconstructed phylogenies with complete taxon sampling (*N* = 20); the green line shows the average LTT plot with 90% of extant species sampled, *N* = 18 (i.e., 10% of taxa have been randomly pruned at time *t* = 0); the dark blue line shows the average LTT plot with 70% species sampled (*N* = 14); the red and orange lines show the effects of ITS at 50% (*N* = 10) and (30% of extant species sampled (*N* = 6) levels, respectively, on the average LTT plot. The inset shows this effect for one of the simulated trees (N°8) with 100 and 50% taxon sampling. **Table 2** gives the *TreePar* median estimates and associated confidence intervals for the timing of the ME event on each set of simulated phylogenies.

**FIGURE 3:**
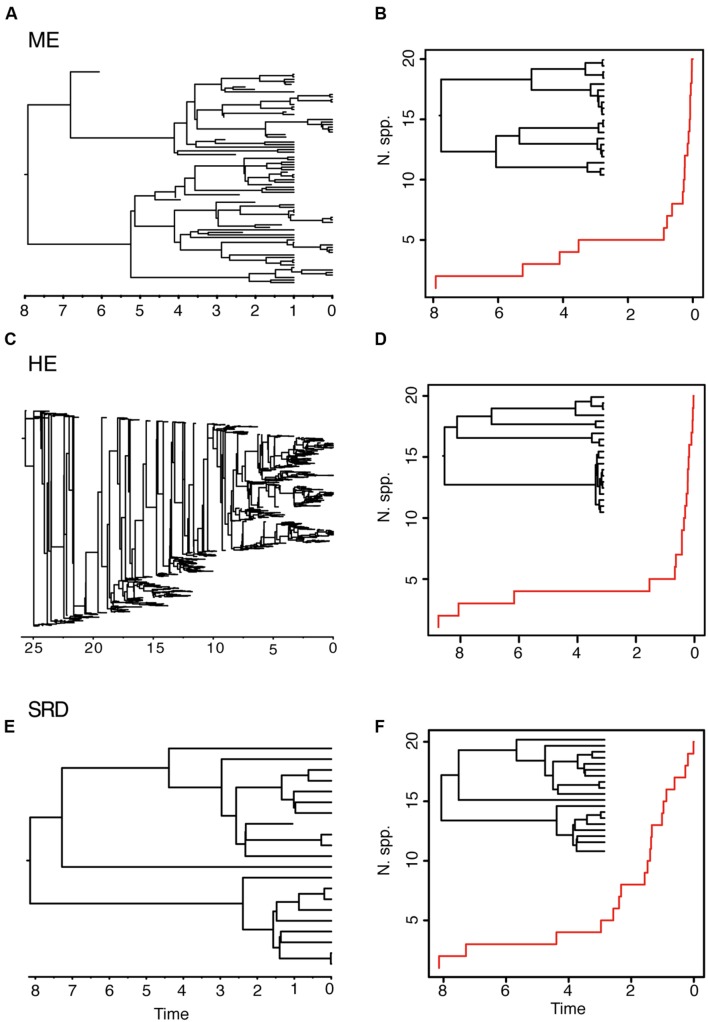
**Different birth–death models can generate reconstructed phylogenies with similar tree shapes.** The figure shows one of 10 complete phylogenies and corresponding reconstructed LTT plot simulated under: **(A,B)** the ME model in **Figures 1G,H**; **(C,D)** the HE model in **Figures 1E,F**; **(E,F)** the “stasis and rate-shift diversification” (SRD) model, simulated as a slow diversification period (λ = 0.2, μ = 0.1, *a* = 0.50), followed by a rate shift or acceleration in diversification rates at time *t* = 3 (λ = 2, μ = 0.01, *a* = 0.005). The inset next to the LTT plot represents the corresponding reconstructed phylogeny. Note that the LTT plot and the reconstructed phylogeny are very similar for the three scenarios; differences may only be fully appreciated in the complete phylogeny (left).

Disentangling the processes leading to an increase in diversification rates is even more difficult because different birth–death models can generate a LTT curve with an upturn in the rate of diversification as the one shown by the BD and HE models (red lines in **Figure 1**). **Figure 1G** represents a phylogeny generated under an “episodic birth–death model” in which a constant-rate BD process is interrupted by a ME event that removes 90% of the original lineages at time *t* = 1 Ma. Similar to the HE model (**Figure 1E**), the reconstructed phylogeny of the ME model shows a “broom-and-handle” shape, with long basal branches and young crown groups (**Figure 1G**). After the ME event, there is a new period of birth–death growth: lineages that escaped the ME diversify, resulting in an artificial upturn in the rate of diversification in the LTT plot (Crisp and Cook, 2009; Antonelli and Sanmartín, 2011). If the ME is random, affecting every lineage with the same probability, it has the effect of leaving major phylogenetic branches intact while pruning the subclades within, resulting in a time interval when origination is smaller than expected (Harvey et al., 1994). For this reason, the LTT plot of a ME model is expected to be sigmoidal, with a growth phase, followed by a plateau, and ending in a rapid increase in diversification rates, which corresponds to the recovery phase (Harvey et al., 1994; Crisp and Cook, 2009; Antonelli and Sanmartín, 2011). In **Figure 1**, the average LTT plot of the ME model (**Figure 1H**) looks flatten and slightly sigmoidal, compared to the more convex shape of the LTT plot in the HE model (**Figure 1F**; see also **Figure 3**). The starting of the recovery phase after the plateau indicates the time of the ME event in the LTT plot (Harvey et al., 1994), and this has been used to identify the geological or climatic event that caused the ME (Crisp and Cook, 2009). Antonelli and Sanmartín (2011), however, suggested that ITS may delay the start of the recovery phase if, by chance, ITS (i.e., akin to a ME event at *t* = 0) fails to sample some of the clades that survived the ME so that the start of the post-ME diversification is pushed forward in time. **Figure 2** shows the average LTT plot for the 10 ME reconstructed phylogenies simulated in **Figure 1H**, but under increasing levels of ITS. As taxon sampling increases, there is an apparent delay in the time of the expected recovery in the LTT plot, though this delay is only noticeable in the average curve with 30% taxon sampling (**Table 2**; the inset shows this pattern for one simulated tree, N° 8). Given the small number of simulations and the large variance around the estimated values, this remains an observation in need of statistical testing.

**Table 2:** Overall accuracy and precision of *TreePar* to estimate the time of the ME event in the ME model for small phylogenies and under increasing levels of ITS.

Sampling	100%	90%	70%	50%	30%
**Time of ME event:**					
Median	1	1	1	1	0.82
95% CI	(0.9–1.01)	(0.9–1.01)	(0.8–1.20)	(1–1)	(0.66–0.94)
MAPE	0.34	1.02	1.08	0.92	1.02
PREC	2.04	4.94	4.78	5.12	4.98

*Sampling: percentage of sampled taxa from 90% of species sampled (ρ = 0.9) to 30% (ρ = 0.3). The simulated time was t = 1 Ma. Time of the ME event: Median and 95% confidence interval around the median (95% CI, Chambers et al., 1983) of the estimated time of the ME event over the 10 simulated ME phylogenies for each ITS level; the command bd.shifts.optim was used without accounting for ITS (ρ = 1). MAPE: Accuracy was measured as the mean absolute percentage error, calculated as MAPE = 1/n^∗^Sum (absolute_value[estimate – simulated]/simulated), where n = number of simulations. PREC: precision was measured as the size of the confidence interval range, relative to the mean estimated parameter value, calculated as PREC = (estimateMax – estimateMin)/mean estimate).*

The ME and the HE models produce slightly different average LTT plots, but distinguishing between the two scenarios might be difficult based on tree shape alone. For example, in **Figure 3**, the LTT plot of the ME and the HE models show a similar sigmoidal shape, with an initial small slope followed by a plateau and the curve getting steeper toward the present (**Figures 3B,D**). The corresponding reconstructed phylogenies (insets) are also very similar, with long stems and young crown clades. The differences between the two scenarios can only be appreciated in the complete phylogenies (**Figures 3A,C**). Furthermore, these two high extinction scenarios can be difficult to distinguish from a model in which a period of low net diversification (i.e., low speciation and extinction rates) is followed by a rapid increase in the speciation rate (**Figures 3E,F**) (Antonelli and Sanmartín, 2011; Stadler, 2011b). The “stasis and rate-shift diversification” (SRD) scenario generates a LTT plot with an initial slow accumulation of lineages and an upturn in the rate of diversification toward the present (**Figure 3F**). The reconstructed phylogeny is also similar to those of ME and HE models, with nodes clustering toward the present (**Figure 3F**, inset). However, the complete phylogeny (**Figure 3E**) shows that in this case the acceleration in diversification rates is the result of an increase in speciation rates, instead of an artifact of high extinction rates. Distinguishing between the ME and SRD models often requires the use of additional information, such as biogeographic and paleontological (fossil record) data (Antonelli and Sanmartín, 2011; Stadler, 2011b).

Both the HE and ME models represent scenarios in which a large number of lineages have been removed in relation to the number that ever existed, but while this is a discrete time event in the ME model, extinction rates are maintained high and constant over time in the HE model. The apparent result in our simulated phylogenies (**Figure 1**) is a higher loss of diversity in the HE model than in the punctual ME model. Complete phylogenies under the HE model are on average older than those under the ME model (**Figures 1** and **3**), and the mean number of extinct taxa in the complete phylogeny is also higher in the HE than in the ME model (see **Table 1**). In fact, the probability of survival to the present of old clades in a reconstructed phylogeny is negatively correlated with the extinction fraction, 1 – *a* (Harvey et al., 1994). This would suggest that severe and episodic ME events are less “damaging” for conserving ancestral diversity than a scenario with HE rates. One could envision the present diversity crisis as the latter scenario, in which extinction rates are kept high relative to speciation rates across a diverse range of organisms. It must be noted, however, that the ME scenario simulated here is rather recent (*t* = 1 Ma); an older ME event (*t* = 20 Ma) results in a much higher number of extinct taxa in the ME complete phylogenies (mean = 195.1, *SD* = 265.76; simulations not shown), though this number is still lower than the one estimated for the HE model (**Table 1**). Again, our example is only illustrative, given the small number of simulations and the large stochasticity around the inferred values (see **Table 1**).

### Estimating Extinction Rates with Diversification Rate Methods

Comparisons between birth–death models have so far been based here on the shape of the reconstructed tree and changes of slope in the LTT plot (**Figures 1–3**). However, since different models can give rise to similar phylogenetic tree shapes (**Figure 3**), methods that compare diversity trajectories, such as the gamma statistic (Pybus and Harvey, 2000), are in general less powerful than direct estimation of birth and death parameters through statistical inference (Morlon, 2014). Likelihood-based diversification rate methods (reviewed in Pyron and Burbrink, 2013; Stadler, 2013; Morlon, 2014), estimate speciation and extinction rates (or more often the relative parameters *r* and *a*) by maximizing the likelihood of the reconstructed tree given the model. Recently, Bayesian approaches have been developed that incorporate uncertainty in the parameter estimation (Bokma, 2008). The use of a likelihood or a Bayesian approach has the advantage of introducing a battery of statistical tests for model choice, such as Likelihood Ratio Tests, the Akaike Information Criterion, or Bayes-Factors comparisons (Morlon, 2014).

Despite this sound statistical framework, inferring extinction rates from timetrees has proven a difficult enterprise (Rabosky, 2010). The accuracy and precision of estimates for the extinction fraction are generally lower than for the net diversification parameter, especially for small trees (Stadler, 2011a). Often, likelihood-based estimates of extinction are unrealistically low (*a*∼ 0; Stadler, 2011a). This stands in contrast with the fossil record, which shows that some clades are currently declining or that they have gone in the past through periods of declining diversity with extinction rates higher than speciation rates (μ > λ) and negative net diversification rates (Quental and Marshall, 2010; Simpson et al., 2011). As seen above, one reason for the low estimates of the extinction fraction is ITS, which, if phylogenetically overdispersed, has the effect of removing the pull of the present (Pybus and Harvey, 2000; Cusimano and Renner, 2010). Corrections for this bias have become standard in diversification rate methods, including for non-random, phylogenetically clustered ITS (Höhna et al., 2011; Cusimano et al., 2012). Error in the estimation of the topology and phylogenetic branch lengths, for example, through incorrect modeling of the DNA substitution data, is another source of bias, as these data form the basis for estimating the extinction fraction. Great effort has gone into correcting for this methodological bias in the last decade (reviewed in Laurin, 2012).

A different reason for the unrealistically low extinction estimates is the simplicity of earlier models, which assumed that diversification rates were constant over time (Nee et al., 1994). This is unlikely for large and old lineages, especially if extinction rates are dependent on environmental factors or standing levels of diversity as in the DDC model (Stadler, 2011a). The last decade has witnessed the derivation of the likelihood function under increasingly complex birth–death models (Stadler, 2013; Morlon, 2014): from the simple BD model (Nee et al., 1994) to rate-variable models introducing discrete or continuous functions for time and diversity-dependency (Paradis, 2004; Rabosky, 2006; Rabosky and Lovette, 2008; Etienne et al., 2012). Recently, Stadler (2011b) derived the likelihood function for an episodic *birth*–*death*–*shift process* in which λ and μ can change at discrete times. Unlike piecewise likelihood methods that consider the phylogeny before and after the rate shift as independent trees (Rabosky, 2006), Stadler’s (2011b) approach uses whole-tree likelihood methods to detect rate shifts (i.e., maximizing the likelihood over the entire phylogeny). This allows the model to account for the pull of the present (Nee et al., 1994), while inferring the number and timing of rate shifts. It is not possible, though, to simultaneously estimate multiple rate shifts; instead, the algorithm uses a greedy approach in which the time of one rate shift is estimated and fixed before estimating the time of the next rate shift (Stadler, 2011b). The model can also infer negative rates of diversification (higher rates of extinction than speciation), and it can be used to estimate the number and intensity of ME events. ME events are modeled as sampling events, points in time in which the standing diversity is reduced by a fraction – controlled by the parameter ρ. Yet, it remains difficult to simultaneously estimate diversification rate shifts and the timing and intensity of ME events due to overparameterization; one of these parameters needs to be fixed; for example, by assuming that μ and λ have remained constant before and after the ME event, or by fixing the sampling intensity of the ME event before inferring the timing and number of rate shifts (Stadler, 2011b). Recently, May et al. (2016) proposed a new Bayesian approach, CoMET implemented in the R package TESS (Höhna et al., 2015), in which temporal rate heterogeneity (diversification rate shifts) and ME events are modeled through Compound Poisson Processes, with the first considered as nuisance parameters in the Bayesian inference. The timing and intensity of the ME can also be assigned informative priors based on paleontological knowledge to avoid overparameterization.

An additional limitation of early birth–death models was the assumption of equal diversification rates across clades. Given that speciation and extinction rates are likely dependent on species-specific biological traits (Ezard et al., 2011), rate homogeneity across clades seems unrealistic. One of the most important advancements in this field came from the work performed by Rabosky et al. (2007), Alfaro et al. (2009), Rabosky (2014), who developed a series of tools to detect rate shifts within a phylogeny that might be indicative of different diversification regimes across clades. MEDUSA (Rabosky et al., 2007; Alfaro et al., 2009) uses an AIC-stepwise approach to sequentially compare a background model of constant birth–death rates against more complex models in which diversification rates are allowed to vary across clades but are assumed constant within each clade. BAMM (Rabosky, 2014) extended this approach to allow time-varying speciation rates within subclades. One advantage of this approach is the possibility to account for ITS by assigning target clades their standing taxonomic richness. Turnover rates, however, are assumed constant over time; only the constancy in the rate of speciation and net diversification is relaxed. Recently, Morlon et al. (2011) derived an analytical likelihood expression for relaxing simultaneously the time-constancy and homogeneity of diversification rates across lineages by allowing speciation and extinction rates to vary across subclades within a phylogeny but also continuously over time. This provided estimates of μ that were more in agreement with the fossil record, supporting waxing-and-waning dynamics in which some clades have rates μ > λ. One drawback is that the target subclades need to be defined *a priori* using taxonomic information, although an AIC-stepwise procedure similar to MEDUSA could be used (Morlon, 2014).

Although analytical derivation of the likelihood of branching times under a given model is undoubtedly the most powerful approach, a common characteristic of all methods reviewed above is that they usually require a large amount of phylogenetic data to reliably quantify extinction rates (Stadler, 2013). Beaulieu and O’Meara (2015) investigated the performance of likelihood-based diversification methods to estimate extinction rates from reconstructed phylogenies, and concluded that even under cases of rate heterogeneity, extinction rates could be estimated from phylogenies of moderate size (*N* ≥ 50), though with large confidence intervals. Laurent et al. (2015) examined the ability of Stadler’s (2011b) episodic birth–death model, implemented in the R package *TreePar*, to detect ME events, and showed that the statistical power of TreePar increased with the size of the phylogeny (*N* > 200–300) and the intensity of the ME event (ρ > 0.2). **Table 2** illustrates the ability of *TreePar* to estimate the timing of the ME event in our very small (20-taxa) reconstructed phylogenies. As found by Laurent et al. (2015), the accuracy and precision of *TreePar* decreased with increasing levels of ITS, and were generally not very high (large MAPE and PREC values, **Table 2**), which agrees with the idea that likelihood-based methods have reduced statistical power for small phylogenies (Stadler, 2011b). A larger study design is needed to properly test this bias.

The ME models simulated in **Figure 1** assume “random” ME, in which the surviving lineages form a random phylogenetic sample of all clades or all lineages have the same probability to get extinct, a “field of bullets” scenario (Laurent et al., 2015). There are, however, two other types of ME: “uniform” extinction or “wanton destruction,” in which for every lowest-level clade, one representative becomes extinct, and “clade” extinction or a “fair game” scenario, in which all members of a clade become extinct (Harvey et al., 1994) or the probability of survival depends on a clade-specific trait (Laurent et al., 2015). *TreePar* seems to perform well in the estimation of random ME events (especially if of high intensity), but power to detect these events decreases in the cases of “clade” and “wanton” extinction (Laurent et al., 2015). Interestingly, MEDUSA could perform well under this type of scenario if it interprets clade extinction as a “lineage-specific” rate shift, a rapid increase in the extinction fraction or a decrease in the net diversification rate in one subclade within the phylogeny. Conversely, the performance of *TreePar* is affected by the presence of rate heterogeneity across lineages, interpreting these clade rate shift events as ME false positives (Laurent et al., 2015). A common finding of these studies is that the number of lineages preceding the ME event must be large (*N* > 100) to be able to distinguish stochastic effects from a true ME event or a rate shift (Stadler, 2011a; Laurent et al., 2015; May et al., 2016). This makes more recent ME events easier to detect than older events, because background extinction has likely removed the signal of the older lineages. It might also explain why it is easier to detect ME events when these are preceded by a period of rapid diversification, i.e., low background extinction rates, for example, in the case of an evolutionary radiation (Crisp and Cook, 2009; Antonelli and Sanmartín, 2011; see also **Figure 1**).

Although analyzing a 20-taxa phylogeny might seem unrealistic, we recently found ourselves in that position when working with species-poor Rand Flora clades (Pokorny et al., 2015). What is the solution in those cases? Sometimes adding an additional layer of information helps. Using only fossil data, even incomplete (Laurin, 2012), or including fossil taxa in a time-calibrated extant phylogeny (Ronquist et al., 2012) may help obtaining more realistic extinction rate estimates (Magallón, 2010), especially if a parameter accounting for differences in the process of fossilization is included in the model (Didier et al., 2012; Silvestro et al., 2014). Tying the variation in diversification rates to changes in external environmental factors is another option (Condamine et al., 2013). More promising are the recently developed trait-dependent diversification models, also known as the state speciation and extinction “SSE” family of models, in which speciation and extinction rates are associated to the evolution of a character or phenotypic trait over the phylogeny (Maddison et al., 2007). In models such as BiSSE (binary-state speciation and extinction), each character state is assigned a separate speciation and extinction parameters, and the character itself is allowed to change over time according to estimated transition rates. Expansion of these models to allow temporal variation (FitzJohn, 2012) truly integrates rate heterogeneity over time and across clades. However, these models have proven even more data-demanding than time-dependent rate models (Davis et al., 2013). One potential solution is to increase the number of data points (branching times) by using phylogenetic data from multiple clades. Since a ME event presumably acts clade-wide, its phylogenetic signature should be imprinted across a diverse range of organismal phylogenies. A hierarchical Bayesian approach in which the time and intensity of the ME event is estimated across clades, while allowing the extinction fraction – dependent on intrinsic biological traits (Jablonski, 2008; Purvis, 2008) – to differ among clades, could be used to detect these large-scale extinction events affecting a region’s biota (May et al., 2016; see below for a similar approach in biogeography).

## Biogeographic Approaches to the Inference of Extinction

### Inferring Geographic Extinction from Spatial Data

**FIGURE 4:**
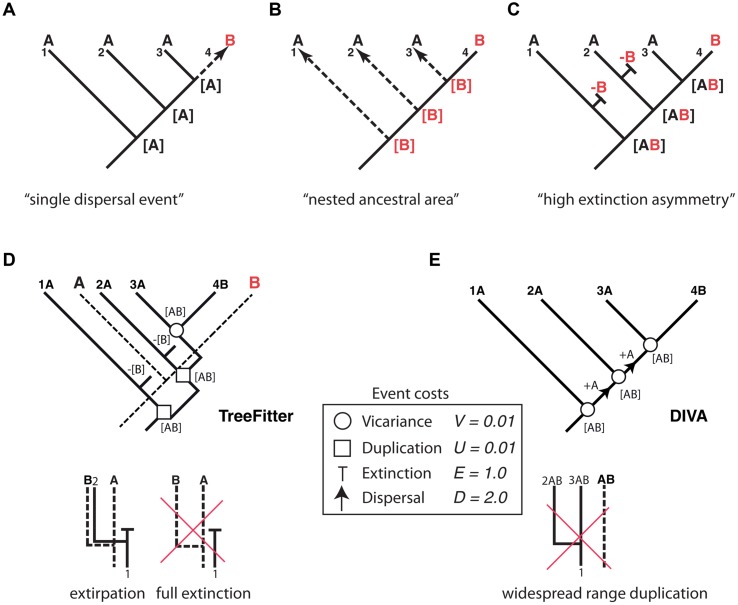
**Effect of differential rates of extinction across areas on the recovery of biogeographic patterns.** One lineage distributed in area B is embedded within a paraphyletic assemblage restricted to area A. Three alternative explanations: **(A)** “Single dispersal event” scenario: the most parsimonious explanation is a single dispersal event to B preceded by successive speciation events within area A; **(B)** “nested ancestral area” scenario (Cook and Crisp, 2005): strong directional dispersal asymmetry favors area B as ancestral; **(C)** “high extinction asymmetry” scenario (Sanmartín et al., 2007): duplication within a widespread distribution with a higher extinction rate in B than in A explains this pattern. **(D)** An event-based reconstruction of the biogeographic pattern in **(A–C)**, using parsimony-based tree fitting (TreeFitter v. 1.2; Ronquist, 2003). Under the default cost assignments [vicariance (*V*) = duplication (*U*) = 0.01; extinction (*E*) = 1; dispersal (*D*) = 2], the “high extinction asymmetry” scenario has a cost of 2*U* + 2*E* + 1*V* = 2.03, only slightly higher than the “single dispersal event” scenario (cost = 2*U* + 1*D* = 2.02), but considerably lower than the “nested ancestral area” scenario (cost = 3*D* = 6.0). Note that extinction in TreeFitter is modeled as extirpation tied to a speciation event; “full extinction” cannot be modeled, because it does not leave observable descendants in the phylogeny. **(E)** Event-based reconstruction of the biogeographic scenario in **(A–C)** using Dispersal-Vicariance Analysis (DIVA); since this method does not allow duplication within widespread ancestors, extinction events are never inferred in the most parsimonious reconstruction (see text for further explanation).

Thus far, we have dealt with lineage extinction, the disappearance of a taxon/taxa within a clade’s phylogeny. Biogeographers, however, are concerned with another dimension of extinction: the spatial. Extinction in biogeography often refers to the disappearance of a species or clade from part of its distributional range (extirpation or range contraction) and more rarely to full extinction, i.e., the complete disappearance of the taxon (Sanmartín, 2012). Paleogeographers such as Bruce Lieberman (Lieberman, 2002, 2005) argued that the process of extinction has an effect on biogeographic reconstructions similar to the one produced by ITS on phylogenies: by removing some of the existing diversity, extinction may lead to inaccurate inferences of past geographic ranges. Biogeographic studies of extant groups that have lived for a very long time and have high extinction rates should thus be avoided (Lieberman, 2002). Meseguer et al. (2015) recently demonstrated that if high extinction rates are coupled with a strong spatial bias – higher extinction rates in one region than in others – it becomes very difficult to reconstruct the correct biogeographic history of an extant clade without additional fossil data. **Figure 4** shows one of these “high extinction asymmetry” scenarios. Given a phylogeny with one lineage distributed in area B nested within a paraphyletic set of lineages occupying area A, the simplest biogeographic explanation – with the least number of ad hoc assumptions – is one in which the lineage was originally present in area A and underwent successive speciation events within this area, followed by one late dispersal to area B (**Figure 4A**). However, in cases of high dispersal asymmetry – in which dispersal from B to A is favored over dispersal from A to B – area B could actually be the original distribution of the lineage, with each species in A arising from independent dispersal events (**Figure 4B**) (Cook and Crisp, 2005). This situation may occur if prevailing wind currents strongly favor directional dispersal from B to A over the opposite direction (Cook and Crisp, 2005). There is, however, a third scenario (**Figure 4C**) that does not require dispersal events. If extinction rates have been historically higher in area B than in area A, successive speciation events of a widespread ancestor distributed in AB, followed by extinction in area B except in one lineage, could explain the nested distribution (Sanmartín et al., 2007). As in Cook and Crisp’s (2005) nested ancestral area scenario, most biogeographic methods will fail to recover this high extinction asymmetry reconstruction, simply because there is not enough information in the phylogeny and present distributions to predict the loss of area B along each terminal branch (Sanmartín et al., 2007).

How has extinction fared in analytical historical biogeography and in particular the high extinction scenario? Parsimony-based “cladistic” biogeographic methods (Nelson and Platnick, 1981; Brooks, 1985) aim to find congruent distribution patterns among organisms as evidence of shared biogeographic history (i.e., vicariance), and treat extinction and dispersal as a source of homoplasy (noise) in biogeographic reconstructions. Since these two processes are specific to single lineages, they might obscure the pattern of biogeographic congruence generated by vicariance unless they are minimized in the reconstructions (Sanmartín, 2012). For the scenario depicted in **Figure 4**, cladistic methods would recover the “single dispersal event” scenario (**Figure 4A**) as the most parsimonious explanation for the observed distribution pattern, since this reconstruction implies the minimum number of extinction (0) and dispersal (1) events.

Event-based biogeographic methods, such as TreeFitter (Ronquist, 2003; Sanmartín et al., 2007) or DIVA (Ronquist, 1997), treat extinction as one of four different types of biogeographic events: vicariance, dispersal, extinction, and duplication (i.e., within-area speciation). Each process is associated with a positive value or “cost” that should be inversely related to its likelihood, and the analysis consists in finding the biogeographic reconstruction with the minimum cost, i.e., the most parsimonious explanation. Simulations (Ronquist, 2003) have shown that in order to maximize the recovery of “phylogenetically constrained” biogeographic patterns in event-based methods – distribution patterns that are conserved along the phylogeny – extinction and dispersal must be assigned higher costs relative to vicariance and duplication. This is because dispersal and extinction imply a change in the distribution of the descendants relative to the ancestral range (addition of a new area or subtraction of one area from the ancestral range), whereas vicariance and duplication imply the full inheritance of the ancestral distribution among the two descendants (Sanmartín et al., 2007). In practice, this implies that dispersal and extinction events are underestimated (minimized) in event-based reconstructions. For example, under default cost assignments (Ronquist, 2003; Sanmartín et al., 2007), TreeFitter will recover the “single dispersal event” scenario, though the cost of the “high extinction asymmetry” scenario is only slightly higher (**Figure 4D**). One reason for this is that in event-based reconstructions, extinction (*E*) is assigned a lower cost relative to dispersal (*E* = 1.0, *D* = 2.0, see **Figure 4D**) in order to minimize the impact of missing areas when analyzing multiple clades (Sanmartín and Ronquist, 2002). Another important constraint of TreeFitter is that extinction is modeled as extirpation tied to speciation, that is, the disappearance of a taxon from part of its distributional range following a cladogenetic event (**Figure 4D**). Full extinction, the disappearance of a taxon from its entire distributional range (**Figure 4D**, right), cannot be inferred with event-based or any parsimony-based method because this type of event (like full dispersal) does not leave any observable descendant in the phylogeny (Ronquist, 2003). **Figure 4E** shows an event-based reconstruction inferred using Dispersal Vicariance Analysis, DIVA (Ronquist, 1997), probably the most popular event-based method. Though extinction is a cost event in DIVA, in practice these events will never be inferred in the most parsimonious reconstruction unless geological constraints are introduced in the cost matrix. This is because, unlike TreeFitter (**Figure 4D**), DIVA does not allow duplication of a widespread range (**Figure 4E**); instead, widespread ranges are divided by vicariance at each speciation node, with subsequent dispersal needed to reconnect the two areas before the next speciation event (Sanmartín, 2012).

### Model-Based Parametric Methods and Asymmetric Extinction

**FIGURE 5:**
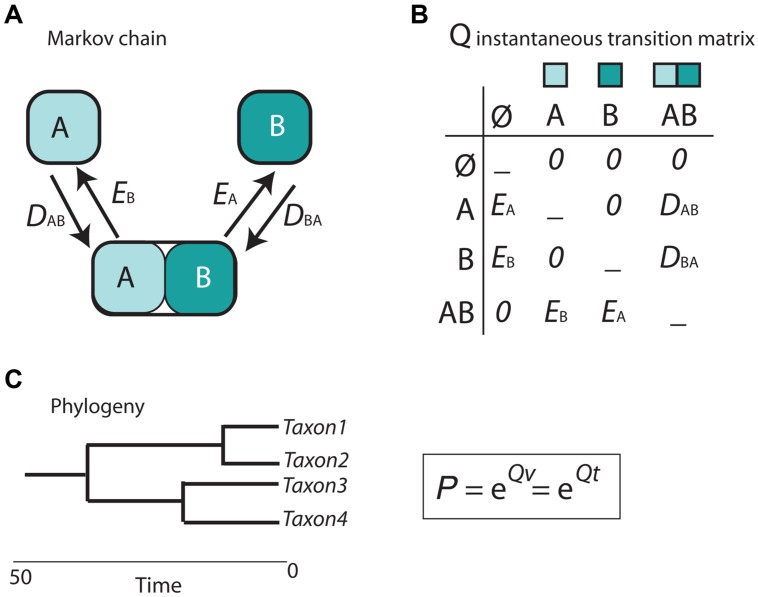
**Scheme depicting the parametric Dispersal-Extinction -Cladogenesis model (DEC). (A)** range evolution is modeled as a continuous-time Markov Chain stochastic process (CTMC) with discrete states, the geographic ranges A, B, and AB; **(B)** transitions or movement between states is governed by an instantaneous transition rate matrix (*Q*), whose parameters are biogeographic processes: dispersal (range expansion, *D*_AB_) and extinction (range contraction, *E*_A_). Notice that direct movement from A to B is not allowed as it will require two instantaneous events: *D*_AB_ + *E*_A_. **(C)** Exponentiating the *Q* matrix as a function of time (*t*) or evolutionary divergence (branch lengths, *v*) gives the probability (*P*) of geographic change along phylogenetic branches.

Cladistic and event-based methods rely on parsimony as the optimization criterion in biogeographic inference. As discussed in Sanmartín (2012), this has several drawbacks, including the difficulty to estimate the rate of biogeographic processes (i.e., the likelihood of different event types) from biogeographic data, or the impossibility to integrate lineage divergence times into the biogeographic reconstruction (Sanmartín, 2012). The main contribution of the parametric or model-based school of biogeography is the introduction of probabilistic models describing the evolution of geographical characters a function of rates of parameters and time (Ree and Sanmartín, 2009; Ronquist and Sanmartín, 2011). In the popular Dispersal-Extinction-Cladogenesis (DEC) model (Ree et al., 2005), implemented in the software Lagrange (Ree and Smith, 2008), extinction (range contraction) and dispersal (range expansion) are modeled as stochastic processes that occur along the branches of a phylogeny (**Figure 5**). The relative rate at which these processes occur is modeled by a continuous-time Markov Chain (CTMC) process with discrete states or geographic ranges (A and B in **Figure 5A**), governed by a matrix of instantaneous transition rates between states (*Q* in **Figure 5B**). By exponentiating this matrix over the branch lengths of the phylogeny, measured in units of time or proportional to evolutionary divergence, it is possible to estimate the probabilities of change among the geographic ranges in the model (**Figure 5C**). Unlike in parsimony-based approaches, tree branches in parametric biogeography inform on both the sequence of ancestor-descendant events (i.e., the tree topology) and the expected amount of biogeographic change: the longer the phylogenetic branch, the higher the probability for biogeographic change and the larger the uncertainty in the ancestral range estimate (Ree and Sanmartín, 2009).

In DEC, extinction is modeled as extirpation or range contraction (*E*_A_), a process that can only remove a single area in an instant of time. Direct dispersal between single areas (from A to B) is assigned a rate of zero in the *Q* matrix (**Figure 5B**) because it would require two instantaneous transition events: a range expansion from A to B (*D*_AB_), followed by extinction in the source area A (*E*_A_). Full lineage extinction is allowed if affecting a single area (**Figure 5B**). In practice, this implies that areas where a taxon is not present could still be part of its ancestral range with low probability, especially if constraints are applied on dispersal rates (Buerki et al., 2011). In recent years, expansions of the DEC model have been developed that include direct movement between single areas (jump dispersal or “founder-event speciation”; Matzke, 2014). The expanded DEC model implemented in the R package BioGeoBEARS (Matzke, 2013) incorporates a free parameter “j” that weights for the probability of jump dispersal versus range expansion at points in the phylogeny. Reconstructed scenarios with DEC + J usually include very low or null extinction estimates compared to DEC-inferred scenarios. One explanation is that extinction in DEC is often estimated along long branches, where loss of areas within widespread distributions, acquired by range expansion, cannot be countered off by cladogenesis (i.e., via vicariance or peripheral isolate speciation). In DEC + J, these extinction events are not inferred because a jump dispersal at the next cladogenetic event is modeled instead.

**FIGURE 6:**
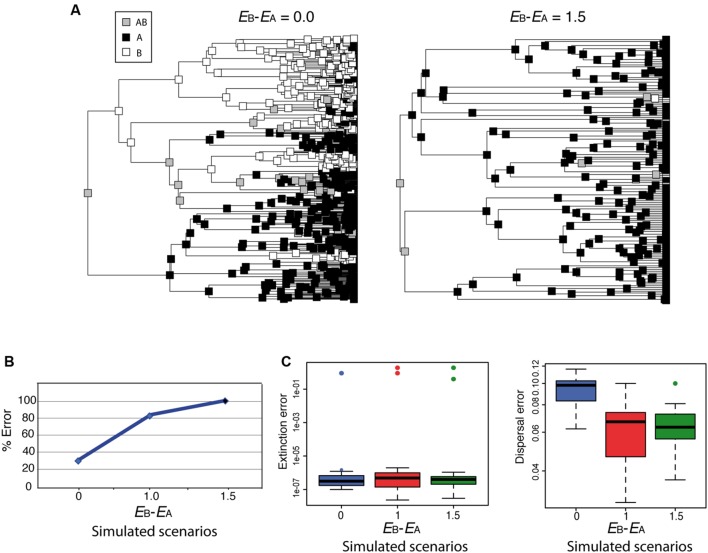
**Effect of differential extinction – different rates of extinction across areas – on ancestral range reconstruction in DEC. (A)** Phylogenies were simulated using a geographic range-dependent diversification model implemented in the software *SimTreeSDD* (Goldberg, 2014); in the left phylogeny, areas A and B were assigned the same extinction rates (*E*_B_ – *E*_A_ = 0.0); in the right phylogeny, extinction rate in B is higher than in A (*E*_B_ – *E*_A_ = 1.5); speciation and transition rates were assumed to be equal between areas (*s*A = *s*B; *D*_AB_ = *D*_BA_); the simulation started with a widespread range AB assigned to the root node. A third, intermediate scenario, not represented here, was simulated with a lower difference in extinction rates (*E*_B_ – *E*_A_ = 1.0). **(B)** Percentage of simulations (of a total of 10 for each scenario) that resulted in wrong inferences by DEC of the root ancestral range. Differential extinction can mislead DEC ancestral inference, especially if there is a large difference in extinction rates between areas. **(C)** DEC estimated rates of extinction (left) and dispersal (right) were considerably lower than the simulated values (colored circles), but the effect was more severe for extinction (see Ree and Smith, 2008).

Although parametric methods such as DEC allow estimating the rate of extinction from biogeographic data, simulations have shown that extinction and dispersal rates are consistently underestimated, and that this is more severe for extinction than for dispersal (Ree and Smith, 2008). How would DEC behave under the high extinction asymmetry scenario depicted in **Figure 4**? **Figure 6** shows two biogeographic scenarios simulated under a trait-dependent diversification model (Goldberg, 2014) in which areas A and B were assigned equal speciation rates (*s*A = *s*B), and the rate of transitioning between states was also assumed equal (*D*_AB_ = *D*_BA_). In **Figure 6A** (left), extinction rates were set equal between areas (*E*_A_ = *E*_B_ = 0.9; *E*_B_– *E*_A_ = 0.0); in **Figure 6A** (right), an asymmetric or differential extinction scenario was modeled with a higher extinction rate in B than in A (*E*_A_ = 0.4, *E*_B_ = 1.9; *E*_B_– *E*_A_ = 1.5). The simulation started with a widespread range AB assigned to the root node. Notice that under the high extinction asymmetry scenario (**Figure 6A**, right), the widespread range AB is lost soon after the initial split, and very few nodal ranges are reconstructed as B compared to the equal-rates extinction scenario (**Figure 6A**, left). Ten phylogenies were simulated under each of these scenarios, and under a third scenario in which the difference in extinction rates between A and B was smaller (*E*_A_ = 0.9, *E*_B_ = 1.9; *E*_B_ – *E*_A_ = 1.0). We then recorded how many times the root ancestral range was correctly reconstructed as AB by DEC. Though our simulation sampling size and design are too small for any statistical significance test, **Figure 6B** suggests that the percentage of wrong reconstructions of the root ancestral range increases in DEC with higher levels of differential extinction between areas. Rates of extinction (**Figure 6C**, left) were also estimated several orders of magnitude lower (between 1e – 7 and 1e – 6) than the original (simulated) values, while this difference was smaller for dispersal rates (**Figure 6C**, right), as observed by Ree and Smith (2008).

The pattern of extinction asymmetry depicted here might appear too extreme but could happen under events of extinction driven by climate change, for example, if one continent was hit harder by global climate cooling (e.g., Pleistocene glaciations) than others (Sanmartín et al., 2001; Meseguer et al., 2015). Unfortunately, for most cases, without additional sources of information it would be difficult to detect the signature of differential extinction in a phylogeny. One possibility is to use range-dependent diversification models, such as the Geographic State Speciation and Extinction model (GeoSSE; Goldberg et al., 2011) to tie range evolution to diversification dynamics. GeoSSE is an extension of the “SSE” family of models to geographic settings, in which extinction and speciation rates are allowed to differ among areas. Unlike BiSSE, however, the evolution of the geographic character in GeoSSE can both affect and be affected by the diversification process. For example, the effective rate of speciation is higher in widespread ancestral lineages (AB) than in lineages endemic to single areas, and, conversely, more extinction events are needed for a widespread lineage to get extinct than for an endemic one (Goldberg et al., 2011). The GeoSSE model can be used to test for historical differences in extinction rates across biogeographic regions: Did taxa living in one area experience higher extinction rates than those living in other geographic regions? Incorporating fossil taxa into the phylogenetic or biogeographic analysis might also improve the accuracy of biogeographic reconstructions, especially when extinction rates have been historically high (Mao et al., 2012; Meseguer et al., 2015). Fossil-informed ecological niche models have been used to distinguish among alternative biogeographic scenarios; for example, by showing that the group under study had a wider geographic distribution in the past and thus its current restricted range should be attributed to ancient extinction rather than a recent long-distance dispersal event (Meseguer et al., 2015).

### Estimating Clade-Wide Extinction Events from Biogeographic Data

**FIGURE 7:**
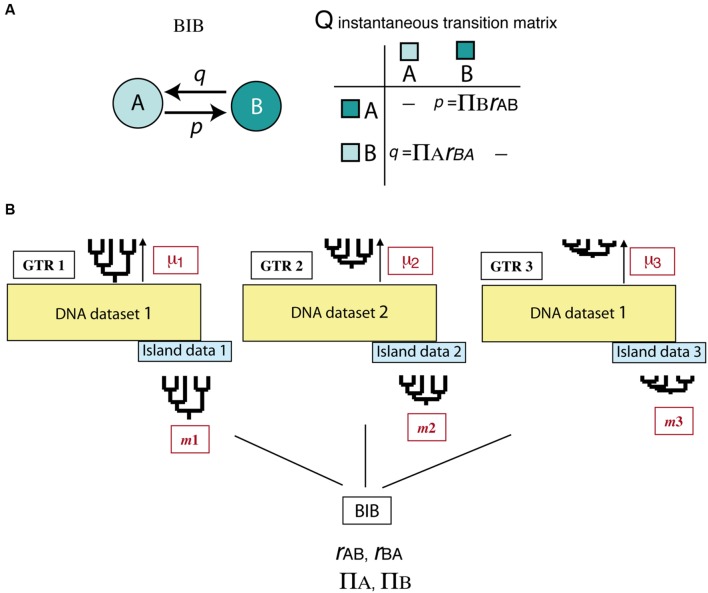
**Bayesian Island Biogeography (BIB) model (Sanmartín et al., 2008). (A)** Geographic evolution is modeled as a CTMC process with discrete states represented by single areas (A, B); unlike DEC, there are no widespread ranges in this model. Transition rates in the *Q* matrix (*p*, *q*) are decomposed into two parameters: the relative rate of dispersal between single areas (*r*_AB_), and the area carrying capacity (π_A_), i.e., the number of lineages in each area at equilibrium. **(B)** Scheme showing the hierarchical Bayesian approach used by BIB to estimate biogeographic rates across a set of unrelated clades that share the same biogeographic region but differ in age of origin, mutation rate, and/or dispersal capabilities. Each clade is allowed to evolve under its own molecular and clock models and to move with a clade-specific dispersal rate by introducing “nuisance” parameters in the model that account for these differences: GTR_1_: clade-specific molecular substitution model; *m*_1_: clade-specific dispersal rate; μ_1_: clade-specific mutation rate. The posterior probability of the biogeographic model parameters (carrying capacities and relative dispersal rates) is estimated across clades while integrating over these differences.

All above-mentioned studies deal with the effects of asymmetric extinction on the phylogeny of a single clade or lineage. Yet, when extinction is driven by abiotic factors, such as climatic change, its signature is probably imprinted across a diverse range of organismal phylogenies. Assessing this effect across multiple clades is a difficult task because it requires separating species-intrinsic factors from extrinsic, environment-linked effects. An interesting avenue of research comes from the implementation of Bayesian hierarchical biogeographic approaches. Inspired by molecular evolutionary models, Sanmartín et al. (2008) developed a Bayesian Island Biogeography (BIB) model that uses a continuous-time Markov Chain process to estimate dispersal rates and area carrying capacities (equilibrium frequencies) from DNA sequences and their geographic locations. Initially developed for island settings, the BIB model was later expanded to continental scenarios (Sanmartín et al., 2010) and independently implemented within a phylogeographic context (Lemey et al., 2009; Bielejec et al., 2014). Two aspects make BIB attractive for modeling historical biogeographic scenarios. One is its mathematical simplicity, based on character evolutionary models, which gives it enough flexibility to fit more complex biogeographic scenarios through integration of abiotic factors or dynamic geological histories (Sanmartín, 2016). For example, unlike DEC, the transition matrix in BIB does not incorporate widespread ranges, and rates of change between states are modeled as “jump dispersal” events, i.e., direct migration between single areas without the need to go through a widespread state (*p* = transition *from* A *to* B, **Figure 7A**). Akin to the nucleotide models used in DNA evolution, transition rates in BIB can be disentangled into the product of two parameters, the relative dispersal rate between areas (*r*_AB_), and the stationary frequency or area carrying capacity (π_A_), which is the number of lineages expected in each area at equilibrium conditions (Sanmartín et al., 2008). Though BIB does not explicitly include a speciation component, the effects of extinction on biogeographic patterns may be modeled through changes in the area carrying capacities (see below). The second advantage of the BIB model is the use of a Bayesian hierarchical inference approach which allows parameters of interest to be estimated jointly across a set of lineages – for example, a group of organisms living in the same region – while other (nuisance) parameters are used to account for differences among lineages in intrinsic biological traits (**Figure 7B**). In particular, the BIB model has been used to estimate common rates of inter-area dispersal and within-area diversification across co-distributed island clades, while accommodating clade-specific differences in age of origin, rate of molecular evolution, and dispersal ability (**Figure 7B**) (Sanmartín et al., 2008).

The original CTMC process implemented in BIB is a time-homogenous process, assuming constancy of rates over time (**Figure 7A**). Recently, Bielejec et al. (2014) proposed a time-heterogeneous CTMC process in which the relative rate of dispersal between areas is allowed to vary across time intervals or “epochs.” Variations in the relative dispersal rate can be used to infer the existence of temporary climatic corridors or dispersal barriers, for example, to test whether historical migration rates among Rand Flora groups decreased after the formation of the Sahara Desert. The most promising approach for the inference

of extinction, however, comes from the implementation of non-stationary Markov Chain models (Blanquart and Lartillot, 2008). In these models, the stationary frequencies of the dispersal process (the carrying capacities in the *Q* matrix, **Figure 7A**) are allowed to change at discrete points in time, with the time and intensity of change estimated jointly from the data. This non-stationary BIB model could be used to detect the signal of MEs, evidenced as a sudden decrease in area carrying capacities, due, for example, to a catastrophic event (e.g., geologic, climatic) that wipes out the biota of a given region (Sanmartín, 2016). The Bayesian statistical inference framework offers the possibility to introduce additional sources of evidence, such as assigning a prior probability on spatial extinction rates based on the geological and climatic stability of a region over time, or a prior probability on background extinction rates that is dependent on a clade’s size, age, or climatic preferences. The probability of survival to the ME (ρ) could also be made dependent on clade-specific characteristics instead of assuming that it impacted equally across lineages (i.e., a fair game scenario). By combining this non-stationary Bayesian model with a range-dependent diversification model describing the geographic evolution of individual clades (e.g., GeoSSE), one could study biotic and abiotic, as well as temporal and spatial factors, influencing extinction rates across multiple organismal phylogenies. These are all exciting avenues of research to be explored in the future.

## Author Contributions

IS designed the study. IS and ASM analyzed the data and co-wrote the article.

## Conflict of Interest Statement

The authors declare that the research was conducted in the absence of any commercial or financial relationships that could be construed as a potential conflict of interest.
