# Supplementary figures and images for: Extinction in Phylogenetics and Biogeography: From Timetrees to Patterns of Biotic Assemblage
Source: Front Genet. 2016 Mar 22;7:35. doi: 10.3389/fgene.2016.00035 (PMC4802293; doi:10.3389/fgene.2016.00035)

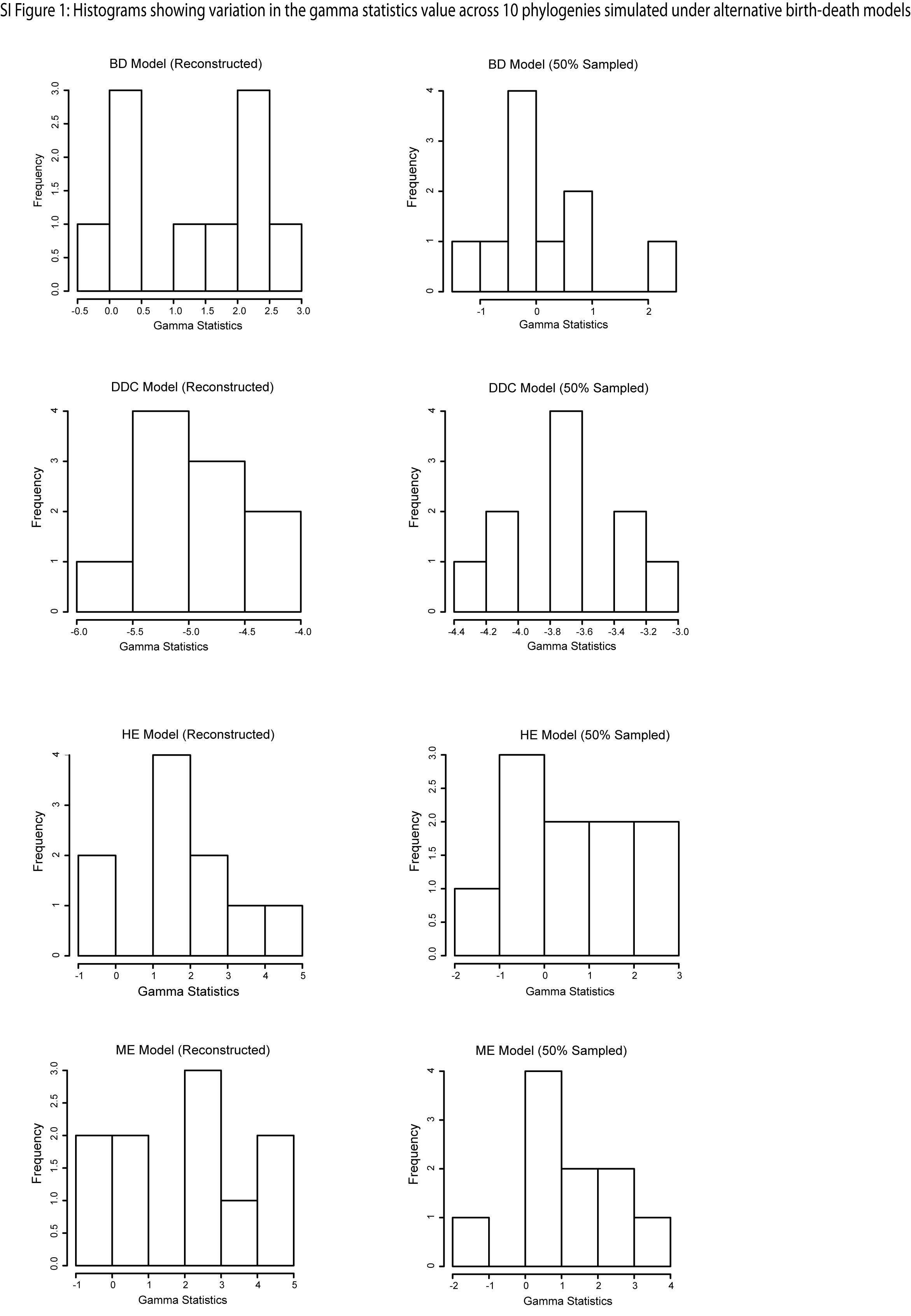

Supplement: Supplementary file 1 [file Image_1.TIF]
